# Virtual Surgical Planning Decreases Operative Time for Isolated Single Suture and Multi-suture Craniosynostosis Repair

**DOI:** 10.1097/GOX.0000000000002038

**Published:** 2018-12-17

**Authors:** Tom W. Andrew, Joseph Baylan, Paul A. Mittermiller, Homan Cheng, Dana N. Johns, Michael S. B. Edwards, Sam H. Cheshier, Gerald A. Grant, H. Peter Lorenz

**Affiliations:** From the * Hagey Laboratory for Pediatric Regenerative Medicine, Division of Plastic Surgery, Department of Surgery, School of Medicine, Stanford University, Stanford, Calif.; † Division of Plastic Surgery, Department of Surgery, School of Medicine, Stanford University, Stanford, Calif.; ‡ Division of Pediatric Neurosurgery, Department of Neurosurgery, School of Medicine, Stanford University, Stanford, Calif.

## Abstract

**Background:**

Cranial vault reconstruction is a complex procedure due to the need for precise 3-dimensional outcomes. Traditionally, the process involves manual bending of calvarial bone and plates. With the advent of virtual surgical planning (VSP), this procedure can be streamlined. Despite the advantages documented in the literature, there have been no case-control studies comparing VSP to traditional open cranial vault reconstruction.

**Methods:**

Data were retrospectively collected on patients who underwent craniosynostosis repair during a 7-year period. Information was collected on patient demographics, intraoperative and postoperative factors, and intraoperative surgical time. High-resolution computed tomography scans were used for preoperative planning with engineers when designing osteotomies, bone flaps, and final positioning guides.

**Results:**

A total of 66 patients underwent open craniosynostosis reconstruction between 2010 and 2017. There were 35 control (non-VSP) and 28 VSP cases. No difference in age, gender ratios, or number of prior operations was found. Blood loss was similar between the 2 groups. The VSP group had more screws and an increased length of postoperative hospital stay. The length of the operation was shorter in the VSP group for single suture and for multiple suture operations. Operative time decreased as the attending surgeon increased familiarity with the technique.

**Conclusions:**

VSP is a valuable tool for craniosynostosis repair. We found VSP decreases surgical time and allows for improved preoperative planning. Although there have been studies on VSP, this is the first large case-control study to be performed on its use in cranial vault remodeling.

## INTRODUCTION

The complex 3-dimensional anatomy of the developing cranial vault makes craniosynostosis reconstruction a challenging surgical procedure. Traditionally, surgical planning and outcomes have been dependent on subjective interpretation of 2-dimensional images, 3-dimensional (3D) cast models, and intraoperative assessments. Although good outcomes are often achieved, the current practice results in variable surgeon-specific outcomes and may result in prolonged intraoperative planning and thus surgical time.

Virtual surgical planning (VSP) through computer-aided design and computer-aided manufacturing techniques provides an alternative workflow to traditional open craniosynostosis surgery.^1,2^ VSP has enabled 3D preoperative planning. With preoperative virtual planning, osteotomies can be modified and tailored during design planning to determine the best design for individual patients. With the creation of the 3D outcome model, results can be more accurately followed over time.

VSP has been shown to be a valuable tool in both preoperative planning and intraoperative decision making for bony craniofacial reconstruction.^3–6^ VSP has been demonstrated to shorten operative time in mandible reconstruction.^5,7^ VSP is already widely integrated in orthognathic surgery with multiple studies demonstrating decreased preoperative planning time; however, there are currently no studies investigating its role in isolated single suture or multi-suture craniosynostosis.^8,9^

Despite the potential advantages documented in the literature, there have been a paucity of case-control studies comparing VSP to traditional open cranial vault reconstruction. No studies that measure the time difference in VSP compared with traditional open craniosynostosis have been published. The literature is largely restricted to small cases series,^6,10–15^ and debate continues regarding production cost and prolonged assembly time.^16^

In this case-control study, we aim to objectively address the advantages and disadvantages of the evolving role of VSP during open craniosynostosis surgery. In particular, we intend to evaluate intraoperative time difference between VSP and traditional surgery as a measurement of surgical efficiency.

## MATERIALS AND METHODS

Prospective evaluation of patients who underwent craniosynostosis surgery via VSP with CAD/CAM was compared with a retrospective control cohort utilizing standard open technique by the senior author. The 2 cohorts were matched on the basis of diagnosis, age, and operation performed. VSP was implemented on October 15, 2015. Control patients were consecutive patients who underwent craniosynostosis surgery without VSP, from July 4, 2010, to October 14, 2015. Cases were defined as those who underwent VSP when VSP was performed and cutting guides with models produced between October 15, 2015, to December 1, 2017. Endoscopic repairs were excluded from the study. Factors evaluated included sex, age, diagnosis, operative procedure, previous craniosynostosis-related operations, number of plates and screws used, intraoperative blood loss, length of operative time, length of preoperative VSP time, length of hospital stay, comorbidities, and postoperative complications. Diagnosis was based on craniosynostosis pattern, which was the same for the VSP and non-VSP groups. Diagnosis was defined as unicoronal, metopic, sagittal, lambdoid, and multiple suture involvement. The operative room time clock was started at skin incision, and the end time was defined as the final suture and before the application of dressings. One patient in the VSP group had an endoscopic bicoronal release without VSP and later underwent open anterior cranial vault reconstruction using VSP. Another patient in the VSP group had staged anterior cranial vault and posterior vault reconstruction, each using VSP. In the non-VSP group, 2 patients had prior vault reconstructive procedures before presenting to the senior surgeon.

The process of computer-assisted surgical planning begins with the acquisition of high-resolution computed tomography scans of the craniofacial skeleton at 1 mm cuts. Individual images are converted to digital imaging and communication in medicine format and forwarded outside for processing [3D Systems (Littleton, Colo.)] for 3-dimensional reconstruction and virtual surgery. An internet-based teleconference is then held between the craniofacial surgery and biomedical engineering teams. The digital imaging and communication in medicine data are used to generate complete 3D images in which virtual osteotomies and bone flap manipulations are performed. One limitation is that the software cannot model the bending of bone, so simulated osteotomies are made to contour the bone flaps for the 3D outcome model. During the web consultation, the final shape is continuously modeled with bone flap repositioning until the optimal shape and cranial index for the individual are obtained. This time is highly influenced by the level of experience of the VSP engineer. The cutting jigs, trimming guides, and final positioning guides are generated based on the virtual surgical planning. The web-conference times were recorded prospectively (Table 1).

**Table 1.**
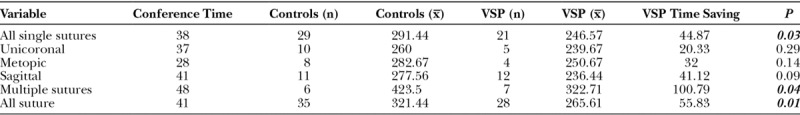
Intraoperative Surgical Time

In this study, surgical procedures were performed with the collaborative efforts of Neurosurgery and Plastic Surgery teams. The cranial vault was accessed through a bicoronal zigzag incision in a subgaleal dissection with care to elevate and preserve a pericranial flap. Suturectomy was performed corresponding to affected suture. For sagittal synostoses, osteotomies were designed from anterior fontanelle to the lambdoid suture synostosis. For lambdoid suture dysmorphology, parietal and occipital bone wedge osteotomies were fashioned. Bilateral total frontal-orbital advancement was designed for metopic and coronal sutures. Cutting guides were placed over the cranium and marked by the craniofacial surgeon. Craniotomy and elevation of bone flaps were performed by the neurosurgeon. The calvarial bone flaps were bent, contoured, and plated by the craniofacial surgeon using the outcome model. For the VSP group, the outcome model was used to guide bone flap and plate contouring. The model was not the absolute determination of final bone and plate shape and contour, which was determined by the craniofacial surgeon’s judgment. All plate systems used were resorbable. Bone grafts were placed and secured. A pericranial flap was utilized. The coronal skin flap was closed, a dressing was applied and the patient was transferred to the PICU.

The only significant change in surgical technique over the study period was a change in resorbable hardware during the final months of VSP study period. The KLS-Martin plate and rivet system was used in the last 2 VSP patients. All other non-VSP and VSP patients had the Synthes resorbable plate and screw system used. No significant changes in the osteotomy cuts, including new or extra osteotomies occurred; however, osteotomy locations were fine-tuned. During the study, the number of guides did not change. One guide was used throughout the study, the cutting guide. The 3D outcome model was used for plate contouring.

Data were statistically analyzed using Microsoft Excel (v16.0); *t* test was used for continuous parametric data, and Fisher’s exact test for categorical data, with *P* < 0.05 indicating statistical significance.

## RESULTS

Patient demographics and operative diagnosis are summarized in Table 2. A total of 66 patients underwent open craniosynostosis reconstruction between July 4, 2010, and December 1, 2017. Three patients, 2 from the control group, and 1 from the VSP group, were excluded due to incomplete postoperative documentation. A total of 63 patients were subdivided into 35 controls (non-VSP) and 28 VSP cases. The average age at the time of surgery for controls was 27.23 months, and 19.75 months for VSP cases, *P* = 0.45. No significant differences in gender and prior operative procedures performed was found between the VSP and control groups.

**Table 2.**
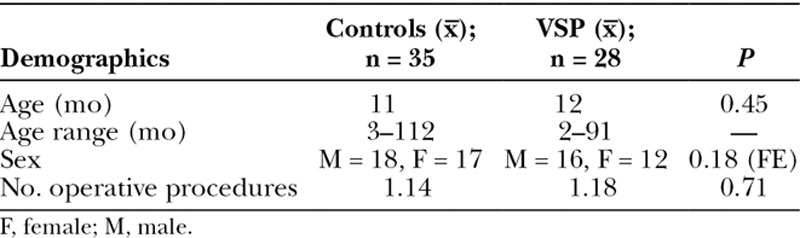
Patient Demographics

A number of intraoperative variables reflecting the complexity of the surgery were measured and results listed in Table 3. A significantly greater number of screws and rivets were used in the VSP group. On average, 100 screws or rivets were used in the VSP group in comparison to 66 screws (no rivets) in the surgical controls (*P* < 0.01). Despite this, no difference in the number of plates opened from original packaging was found (4.89, controls and 4.39, VSP). The number of plates cut from an original package and placed across osteotomies was not recorded in either the VSP or non-VSP group. Intraoperative blood loss was comparable in both groups, 172.21 ml and 182.86 ml for controls and VSP cases, respectively. Despite the similarities in operative complexity, VSP cases stayed in hospital significantly longer than control patients, 5.39 days versus 3.97 days (*P* < 0.01), respectively. In the VSP group, 2 cases of failed extubation resulting in discharge on postoperative day 10 occurred. Despite data adjustments for these clinical outliers, length of hospital stay remained statistically significant. Postoperative complications were similar. One patient in the VSP group and 1 patient in the control group required elevation of the skin flap and debridement due to abscess formation. One VSP patient required revision cranial vault reconstruction for hydrocephalus and ventriculostomy placement, which was not directly related to the VSP.

**Table 3.**
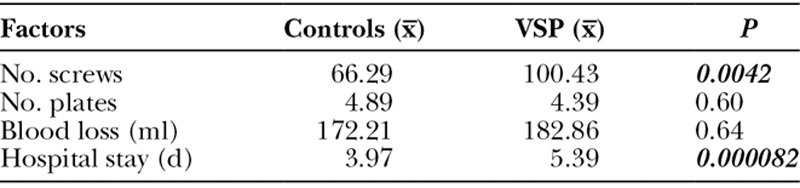
Intraoperative and Postoperative Factors

Operative time was significantly lower in the VSP group compared with non-VSP controls. The operative time for all procedures was on average 321.44 minutes in the control group and 265.61 in the VSP cases (*P* = 0.01). The true impact of VSP on the surgeon includes the preoperative planning time plus operative time. The mean operative time plus preoperative planning session time was 306.29 minutes in the VSP group. We were unable to accurately calculate the preoperative planning time for the non-VSP group. A trend for time-savings in VSP group was present, but without statistical significance. Interestingly, both single suture and multiple suture intraoperative time was significantly less in the VSP group. The average operative time for the single suture VSP group was 44.87 minutes shorter when compared with the control group (*P* = 0.03). Similarly, multiple suture operative time was 100.79 minutes shorter in the VSP group (*P* = 0.04). On assessment of individual suture subtype, no significant difference in operative time between the 2 groups was observed due to loss of statistical power.

**Fig. 1.**
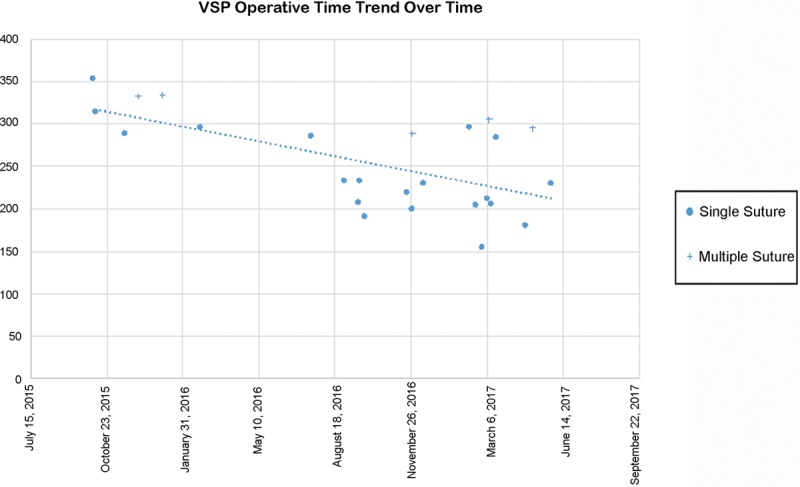
Graph demonstrating trend of VSP operative time over time.

Figure 1 displays the trend in VSP surgical time (minutes) against the date of surgery. A linear regression plot demonstrates a reduction in surgical time over the course of its use. This is thought to be attributed to the adjustment from initial learning to increased proficiency with the technique and benefit of immediate contouring of bone flaps with plate placement as they are harvested.

## DISCUSSION

VSP is a valuable tool for educational and clinical applications. It establishes a reference for surgical goals and comparative postoperative assessment. VSP is performed with the patient’s actual anatomy and provides an unlimited virtual environment for achieving the desired morphological outcomes. The osteotomies and bone movements are designed by the craniofacial surgeon. The software engineer simulates the osteotomies and builds the cutting guides. When designing the osteotomies and bone movements, the surgeon’s knowledge of the underlying anatomy, pathology, and expected outcome is an essential part of the successful use of VSP. Once the plan is finalized, the guides and models help to enable a precise replication of the plan for the operation.

**Fig. 2.**
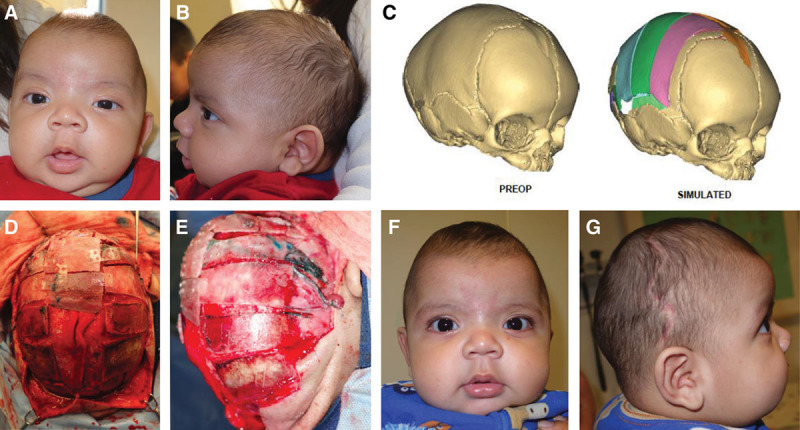
Sagittal craniosynostosis. A and B, Preoperative clinical photographs. C, Cranial vault reconstruction is performed digitally. D and E, An intraoperative guide or model is used to achieve the desired cranial vault shape. F and G, Postoperative clinical result.

**Fig. 3.**
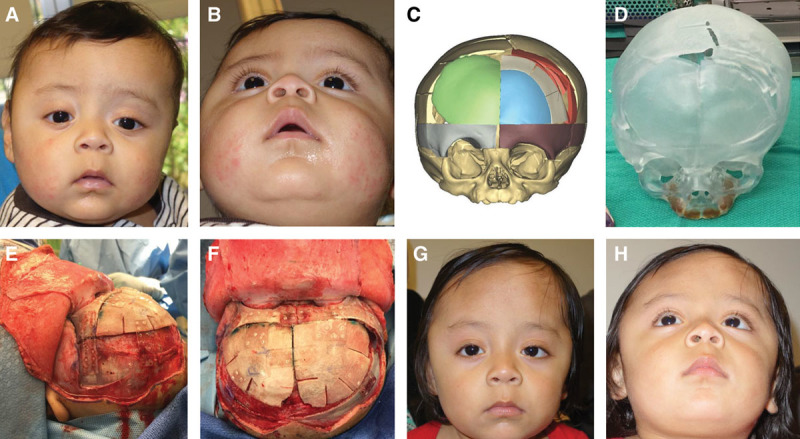
Right unicoronal craniosynostosis. A and B, Preoperative clinical photograph. C, Cranial vault reconstruction is performed digitally. D, A stereolithographic model is 3D printed to show the planned reconstruction. E and F, An intraoperative guide or model is used to achieve the desired cranial vault shape. G and H, Postoperative clinical result.

In this study of patients receiving open craniosynostosis reconstructive surgery with the preoperative and intraoperative assistance of VSP, surgical times were shorter with the use of VSP. We observed a reduction in intraoperative time in patients with single and multiple suture involvement. The current literature supports the role of 3D planning in surgical accuracy, creativity, and reproducibility in craniomaxillofacial surgery.^17–20^ In combination with this, our findings suggest that VSP enhances surgical efficiency (Figs. 2, 3).

Our study demonstrates that operative time was reduced by 45 minutes in single suture patients and 101 minutes in multiple suture involvement. Our findings are congruent with prior studies that have demonstrated that 3-dimensional analysis and surgical planning can reduce surgical time. Khechoyan et al.^15^ compared frontal orbital advancement for standard technique (no modeling) versus modeling using aided CAD/CAM supraorbital bandeau templates. This study used a standardized bandeau based on age and did not include a cutting guide, a 3D model in the OR, did not include full cranial vault osteotomies, and was not patient specific. This study did also found decreased operative time and improved forehead contour outcome compared with the standard technique. An opinion survey reported benefits for mandibular reconstruction using stereolithographic 3-dimensional printing modeling technology.^21^ Emmez et al.^6^ have demonstrated that the use of 3D modeling in mandibular reconstruction can reduce operating time by 2 hours. Similarly, Cohen et al.^7^ described a reduction of 20% median time saving in expended operating room and anesthesia time for a wide variety of procedures using 3D models. However, these data were formulated from an opinion survey of 38 surgeons and failed to comment on craniosynostosis. Despite the limitation of documented evidence, VSP was generally considered to be beneficial in craniosynostosis with multiple suture involvement. VSP use in single suture synostosis is more contentious. However, we have been able to demonstrate that VSP can reduce surgical time in single suture patients with strong statistical significance. This supports prior research on the benefits of VSP for cranial vault remodeling. Emmez et al.^6^ demonstrate improvements in postoperative cranial indices and intracranial volume with the use of VSP in brachycephalic patients.

Our findings broaden the current application and provide empirical evidence in establishing clinical standards in current practice. We hypothesize that VSP reduces surgical operative time in 3 ways: (1) The cranial vault is partially reconstructed ex-vivo using the 3D calvarial outcome model as a guide, which allows the plastic surgeon to begin contouring and plating while the neurosurgeon is still lifting bone flaps and the 2 teams work in parallel. (2) Implants/plates are contoured intraoperatively to the outcome 3D model simultaneously other plating is done on the patient. This is most relevant on the multi-suture cases. (3) The planning phase, which consists of type, extent and magnitude of correction with osteotomy location determination, is largely addressed before the operation.

As a new surgical technique, we observed an initial training period, resulting in an increase in surgical time before the surgeon starts improving his proficiency with the revised operative steps and later decreasing surgical times. We found that, initially, VSP was not faster than traditional methods. However, in the period of less than 2-years, operative time decreased to less than that of traditional methods. These observations in the learning curve have been observed in other surgical techniques, such as laparoscopic surgery, which is now one of the foundations in the surgeon’s armamentarium.^22^ The craniofacial surgeon has 10 years of experience before the beginning of the study, and despite a learning curve continuing for all surgeons we believe this is not as steep as for a junior surgeon. We found VSP total case time (planning plus OR) was no longer for single and was shorter for multiple suture. We expect time savings to improve as VSP technology improves.

Our results found a longer hospital stay in patients who underwent VSP. However, VSP had no effect on postoperative complications, operative blood loss, complexity of surgery, or any other indicators for delayed discharge. We hypothesize that our longer hospital stay is due to current trends in craniosynostosis postoperative management by our team over the last 7 years, such as keeping the patient longer for observation and from a transition to the neurosurgical team from the plastic surgery team managing the patient postoperatively in our center. In addition, we have noted an increased travel distance for families, which tends to prolong hospitalization for discharge planning purposes in our center. However, we are not able to determine if travel time after discharge impacted the decision to discharge in this study.

Limitations to this study include its retrospective nature of the control group, single craniofacial surgeon, and small sample size. Another source of bias related to the employment of VSP and ongoing prospective study to which the surgeon could not be blinded in the setting of measurement of timing is present. However, before this study the aim was to finish the operation as fast as possible. In addition, we believe the time saving of VSP is largely from 2 independent teams working in parallel on 2 different aspects of the operation rather than in series, which was not done before having outcome models. Strengths of this study include the prospective nature of the experimental group. Use of single surgeon was informative in that it helped to clearly discern the learning curve associated in adopting this new surgical technique. Also, although our sample size is small, at the time of publication this is the largest series of VSP patients undergoing calvarial vault reconstruction for craniosynostosis published to date. Future studies will determine accuracy in outcome by assessing need for revision surgery and bone displacement from predicted outcome. With a predicted outcome model, long-term outcomes will be more readily attributable to bone position at the time of initial reconstruction. Our center, as well as many others, do not obtain immediate postoperative CT scans. The 3D model serves as a functional postoperative CT.

This is the first case control study evaluating the efficiency of virtual surgical planning for craniosynostosis repair. We demonstrate significant reductions in intraoperative time with the use of VSP in all craniosynostosis patients. VSP cannot replace the surgeon’s clinical judgment and technical skills in craniosynostosis surgery. However, when appropriately utilized, VSP can formulate precise surgical outcome while reducing operative time and possibly reducing the need for late revision surgery.

## References

[1] Bly RA Chang SH Cudejkova M Computer-guided orbital reconstruction to improve outcomes. JAMA Facial Plast Surg. 2013;15:113 120. 23306963 10.1001/jamafacial.2013.316 PMC5951614

[2] Hierl T Arnold S Kruber D CAD-CAM-assisted esthetic facial surgery. J Oral Maxillofac Surg. 2013;71:e15 e23. 23099224 10.1016/j.joms.2012.08.020

[3] Chopra K Manson PN Gastman BR Stereolithographic modeling in reconstructive surgery of the craniofacial skeleton after tumor resection. Plast Reconstr Surg. 2012;129:743e 745e. 10.1097/PRS.0b013e318245e765 22456402

[4] Gerstle TL Ibrahim AM Kim PS A plastic surgery application in evolution: three-dimensional printing. Plast Reconstr Surg. 2014;133:446 451. 24469175 10.1097/01.prs.0000436844.92623.d3

[5] Mazzoni S Marchetti C Sgarzani R Prosthetically guided maxillofacial surgery: evaluation of the accuracy of a surgical guide and custom-made bone plate in oncology patients after mandibular reconstruction. Plast Reconstr Surg. 2013;131:1376 1385. 23714798 10.1097/PRS.0b013e31828bd6b0

[6] Emmez H Küçüködük I Börcek AO Effectiveness of skull models and surgical simulation: comparison of outcome between different surgical techniques in patients with isolated brachycephaly. Childs Nerv Syst. 2009;25:1605 1612. 19575208 10.1007/s00381-009-0939-y

[7] Cohen A Laviv A Berman P Mandibular reconstruction using stereolithographic 3-dimensional printing modeling technology. Oral Surg Oral Med Oral Pathol Oral Radiol Endod. 2009;108:661 666. 19716728 10.1016/j.tripleo.2009.05.023

[8] Steinhuber T Brunold S Gärtner C Is virtual surgical planning in orthognathic surgery faster than conventional planning? A time and workflow analysis of an office-based workflow for single- and double-jaw surgery. J Oral Maxillofac Surg. 2018;76:397 407. 28826783 10.1016/j.joms.2017.07.162

[9] Wrzosek MK Peacock ZS Laviv A Comparison of time required for traditional versus virtual orthognathic surgery treatment planning. Int J Oral Maxillofac Surg. 2016;45:1065 1069. 27102289 10.1016/j.ijom.2016.03.012

[10] Fisher M Medina M 3rd Bojovic B Indications for computer-aided design and manufacturing in congenital craniofacial reconstruction. Craniomaxillofac Trauma Reconstr. 2016;9:235 241. 27516839 10.1055/s-0036-1584391 PMC4980136

[11] Seruya M Borsuk DE Khalifian S Computer-aided design and manufacturing in craniosynostosis surgery. J Craniofac Surg. 2013;24:1100 1105. 23851748 10.1097/SCS.0b013e31828b7021

[12] Mardini S Alsubaie S Cayci C Three-dimensional preoperative virtual planning and template use for surgical correction of craniosynostosis. J Plast Reconstr Aesthet Surg. 2014;67:336 343. 24333232 10.1016/j.bjps.2013.11.004

[13] Chopra K Folstein MK Manson PN Complex craniofacial reconstruction using stereolithographic modeling. Ann Plast Surg. 2014;72:59 63. 23388241 10.1097/SAP.0b013e3182583f00

[14] Levine JP Patel A Saadeh PB Computer-aided design and manufacturing in craniomaxillofacial surgery: the new state of the art. J Craniofac Surg. 2012;23:288 293. 22337427 10.1097/SCS.0b013e318241ba92

[15] Khechoyan DY Saber NR Burge J Surgical outcomes in craniosynostosis reconstruction: the use of prefabricated templates in cranial vault remodelling. J Plast Reconstr Aesthet Surg. 2014;67:9 16. 24090723 10.1016/j.bjps.2013.09.009

[16] Mendez BM Chiodo MV Patel PA Customized “in-office” three-dimensional printing for virtual surgical planning in craniofacial surgery. J Craniofac Surg. 2015;26:1584 1586. 26106998 10.1097/SCS.0000000000001768

[17] Steinbacher DM Three-dimensional analysis and surgical planning in craniomaxillofacial surgery. J Oral Maxillofac Surg. 2015;73:S40 S56. 26608154 10.1016/j.joms.2015.04.038

[18] Shah A Patel A Steinbacher DM Simulated frontoorbital advancement and intraoperative templates enhance reproducibility in craniosynostosis. Plast Reconstr Surg. 2012;129:1011e 1012e. 10.1097/PRS.0b013e31824effa7 22634677

[19] Diluna ML Steinbacher DM Simulated fronto-orbital advancement achieves reproducible results in metopic synostosis. J Craniofac Surg. 2012;23:e231 e234. 22627442 10.1097/SCS.0b013e31824de612

[20] Pfaff MJ Steinbacher DM Plastic surgery applications using three-dimensional planning and computer-assisted design and manufacturing. Plast Reconstr Surg. 2016;137:603e 616e. 10.1097/01.prs.0000479970.22181.53 26910704

[21] Erickson DM Chance D Schmitt S An opinion survey of reported benefits from the use of stereolithographic models. J Oral Maxillofac Surg. 1999;57:1040 1043. 10484104 10.1016/s0278-2391(99)90322-1

[22] Tekkis PP Senagore AJ Delaney CP Evaluation of the learning curve in laparoscopic colorectal surgery: comparison of right-sided and left-sided resections. Ann Surg. 2005;242:83 91. 15973105 10.1097/01.sla.0000167857.14690.68 PMC1357708

